# Supplementary material for: The Impact of Changes in the Intake of Fiber and Antioxidants on the Development of Chronic Obstructive Pulmonary Disease
Source: Nutrients. 2021 Feb 10;13(2):580. doi: 10.3390/nu13020580 (PMC7916350; doi:10.3390/nu13020580)
Supplement: Supplementary file 1 [file nutrients-13-00580-s001.pdf]

Supplementary Table S1. Baseline characteristics of the study population according to the smoking status at the 5-year follow-up.

Characteristics	Total<br>*n* = 1373	Nonsmoker<br>*n* = 973	Former and Current Smoker *n* = 400	*p*-Value
Age, years	57.62 (0.21)	57.82 (0.25)	57.18 (0.39)	0.173
Males, *n* (%)	467 (34.01)	109 (11.20)	358 (89.50)	<0.001
BMI, kg/m^3	23.22 (0.08)	22.72 (0.09)	24.42 (0.15)	<0.001
AC, cm	82.26 (0.24)	80.12 (0.27)	87.45 (0.40)	<0.001
Pack years, years	28.58 (0.92)	0	28.58 (0.92)	<0.001
Respiratory diseases history, *n* (%)	95 (6.02)	59 (5.97)	36 (9.00)	0.051
TB	68 (4.95)	40 (4.11)	28 (7.00)	
COPD	3 (0.02)	0	3 (0.75)	
Asthma	27 (1.97)	21 (2.16)	6 (1.50)	
Respiratory symptom	510 (37.14)	355 (36.49)	155 (49.26)	0.430
DOE	423 (30.81)	297 (30.52)	126 (31.5)	
Phlegm	61 (4.44)	43 (4.42)	18 (4.50)	
Cough	50 (3.64)	30 (3.08)	20 (5.00)	
Wheezing	36 (2.62)	23 (2.36)	13 (3.25)	
Regular exercise, *n* (%)	883 (64.3)	633 (62.63)	250 (40.44)	0.356
Laboratory finding				
WBC (x 10^3/ul)	5.31 (0.04)	5.16 (0.04)	5.82 (0.08)	<0.001
CRP (mg/L)	0.10 (0.004)	0.08 (0.004)	0.13 (0.010)	<0.001
New airflow limitation	43 (3.13)	11 (1.13)	32 (8.00)	<0.001
Spirometry				
FVC (L)	3.49 (0.02)	3.20 (0.02)	4.18 (0.03)	<0.001
FVC (% of predicted value)	93.83 (0.25)	94.48 (0.30)	92.24 (0.47)	0.884
FEV_1 (L)	2.74 (0.01)	2.55 (0.01)	3.21 (0.02)	<0.001
FEV_1 (% of predicted value)	94.12 (0.26)	94.80 (0.31)	92.45 (0.47)	0.589
FEV_1/FVC	78.84 (0.14)	79.69 (0.16)	76.78 (0.27)	0.015

All values are expressed as a mean (standard error), unless otherwise stated. Abbreviation: BMI, body mass index; AC, abdominal circumference; TB, tuberculosis; COPD, chronic obstructive pulmonary disease; DOE, dyspnea on exertion; WBC, white blood cell count; CRP, C-reactive protein; FVC, forced vital capacity; FEV_1, forced expiratory volume in one second.

Supplementary Table S2. Dietary fiber and nutrient intake according to the presence of a new airflow limitation and smoking status at the 5-years follow-up (2017).

Nutrients	Nonsmoker, *n* = 973		*p*-Value	Former and Current Smoker, *n* = 400		*p*-Value
	Airflow limitation (+) *n* = 11	Airflow limitation (−) *n* = 962		Airflow limitation (+) *n* = 32	Airflow limitation (−) *n* = 368	
Fiber, g	5.68 (0.07)	6.43 (0.05)	0.007	6.45 (0.30)	6.24 (0.07)	0.444
Vitamin A, RE	778.71 (13.66)	811.06 (8.79)	0.697	848.97 (57.67)	801.68 (13.98)	0.348
Vitamin B1, mg	0.91 (0.01)	0.94 (0.01)	0.713	1.04 (0.05)	1.04 (0.01)	0.195
Vitamin B2, mg	1.01 (0.01)	0.99 (0.01)	0.760	1.20 (0.03)	1.06 (0.01)	0.013
Niacin, mg	11.89 (0.18)	12.23 (0.10)	0.724	16.50 (0.63)	15.21 (0.19)	0.054
Vitamin B6, mg	1.60 (0.02)	1.64 (0.01)	0.687	1.94 (0.07)	1.86 (0.02)	0.255
Folic acid, ug	221.22 (3.04)	251.22 (2.10)	0.005	255.71 (11.75)	246.30 (3.15)	0.402
Vitamin E, mg	12.10 (0.18)	12.91 (0.11)	0.439	14.92 (0.64)	13.80 (0.18)	0.082
Vitamin C, mg	94.63 (2.44)	118.07 (2.00)	0.215	103.37 (10.49)	103.45 (2.49)	0.994
Protein, g	58.01 (0.76)	58.74 (0.42)	0.855	75.91 (3.06)	69.45 (0.78)	0.021
Carbohydrate, g	210.36 (2.44)	221.62(1.67)	0.475	250.82 (4.39)	242.39 (1.48)	0.349
Lipid, g	42.73 (0.74)	42.91 (0.38)	0.960	57.68 (1.52)	50.78 (0.36)	0.011
Cholesterol, mg	357.47 (8.24)	323.24 (4.36)	0.407	385.97 (16.73)	363.66 (3.965)	0.464
Total calories	1693.89 (9.36)	1653.43 (5.38)	0.583	2025.74 (29.35)	2005.81 (9.85)	0.564
	Difference from the baseline (2012)					
Fiber, g	−3.72 (0.08)	−2.02 (0.06)	0.002	−2.74 (0.34)	−2.18 (0.09)	0.065
Vitamin A, RE	−403.87 (16.42)	−268.84 (10.55)	0.176	−389.41 (54.82)	−332.19 (17.20)	0.345
Vitamin B1, mg	−0.34 (0.02)	−0.17 (0.01)	0.059	−0.26 (0.03)	−0.21 (0.01)	0.372
Vitamin B2, mg	−0.20 (0.02)	−0.16 (0.01)	0.596	−0.21 (0.03)	−0.20 (0.01)	0.780
Niacin, mg	−4.82 (0.19)	−2.92 (0.11)	0.067	−3.48 (0.30)	−2.97 (0.10)	0.454
Vitamin B6, mg	−0.59 (0.02)	−0.43 (0.01)	0.202	−0.60 (0.03)	−0.48 (0.01)	0.148
Folic acid, ug	−137.07 (22.17)	−71.71 (2.34)	0.003	−103.34 (11.25)	−85.59 (3.76)	0.179
Vitamin E, mg	−4.21 (0.20)	−2.33 (0.12)	0.103	−2.95 (0.34)	−2.82 (0.11)	0.859
Vitamin C, mg	−87.84 (2.98)	−35.78 (2.39)	0.021	−56.25 (4.40)	−43.26 (2.06)	0.237
Protein, g	−14.97 (0.77)	−11.03 (0.44)	0.114	−12.45 (2.70)	−11.44 (0.80)	0.720
Carbohydrate, g	−41.36 (2.64)	−33.37 (1.75)	0.630	−28.66 (4.11)	−29.49 (1.56)	0.933
Lipid, g	−7.33 (0.76)	−4.39 (0.40)	0.436	−4.08 (1.23)	−5.17 (0.37)	0.697
Cholesterol, mg	84.09 (8.43)	17.68 (4.44)	0.114	4.82 (15.71)	20.72 (4.08)	0.609
Total calories	−56.27 (9.95)	−41.92 (1.31)	0.248	−79.82 (8.65)	−72.29 (1.43)	0.619

All values are expressed as a mean (standard error), unless otherwise stated. Abbreviation: RE, retinol equivalents.

Supplementary Table S3. Clinical characteristics according to sex at the 5-year follow-up.

Characteristics	Total $n = 1439$	Male $n = 517$	Female $n = 922$	p-Value
Age, years, n (%)	57.51 (0.21)	58.15 (0.34)	57.15 (0.26)	0.020
BMI, kg/m^3	23.27 (0.08)	24.65 (0.12)	22.49 (0.09)	<0.001
AC, cm	82.46 (0.23)	88.34 (0.31)	79.13 (0.26)	<0.001
Smoking history †, n (%)				<0.001
Nonsmoker	974 (67.69)	109 (21.08)	865 (93.82)	
Former smoker	314 (21.82)	279 (53.97)	35 (3.80)	
Current smoker	151 (10.49)	129 (24.95)	22 (2.39)	
Pack years, years	28.86 (0.88))	31.10 (0.92)	11.63 (1.32)	<0.001
Respiratory diseases history, n (%)	101 (7.02)	47 (9.09)	54 (5.86)	0.021
TB	71 (4.93)	33 (6.38)	38 (4.12)	
COPD	4 (0.28)	4 (0.77)	0 (0.00)	
Asthma	29 (2.02)	12 (2.32)	17 (1.84)	
Respiratory symptom	537 (37.32)	196 (37.91)	341 (36.98)	0.727
DOE	447 (31.06)	161 (31.14)	286 (31.02)	
Phlegm	65 (4.52)	27 (5.22)	38 (4.12)	
Cough	52 (3.61)	24 (4.64)	28 (3.04)	
Wheezing	38 (2.57)	20 (3.87)	18 (1.95)	
Regular exercise, n (%)	925 (64.28)	337 (65.18)	588 (63.77)	0.611
Laboratory finding				
WBC (x 10^3/ul)	5.33 (0.04)	5.72 (0.07)	5.12 (0.05)	<0.001
CRP (mg/L)	0.10 (0.004)	0.12 (0.008)	0.09 (0.005)	<0.001
Glucose (mg/dL)	102.84 (0.61)	109.23 (1.28)	99.25 (0.59)	<0.001
LDL (mg/dL)	125.40 (0.92)	121.09 (1.53)	127.81 (1.14)	<0.001
HDL (mg/dL)	61.03 (0.45)	53.97 (0.66)	64.99 (0.55)	<0.001
New airflow limitation	48 (3.34)	41 (7.93)	7 (0.08)	<0.001
Spirometry				
FVC (L)	3.51 (0.02)	4.26 (0.02)	3.09 (0.01)	<0.001
FVC (% of predicted value)	93.74 (0.25)	91.38 (0.41)	95.07 (0.32)	<0.001
FEV_1 (L)	2.75 (0.01)	3.26 (0.02)	2.47 (0.01)	<0.001
FEV_1 (% of predicted value)	93.96 (0.25)	92.09 (0.41)	95.01 (0.32)	<0.001
FEV_1/FVC	78.75 (0.14)	76.42 (0.22)	80.06 (0.16)	<0.001

† Calculated among smokers. All values are expressed as a mean (standard error), unless otherwise stated. Abbreviation: BMI, body mass index; AC, abdominal circumference; TB, tuberculosis; COPD, chronic obstructive pulmonary disease; DOE, dyspnea on exertion; WBC, white blood cell count; CRP, C-reactive protein; LDL, low density lipoprotein cholesterol; HDL, high density lipoprotein cholesterol; TG, triglyceride; FVC, forced vital capacity; FEV1, forced expiratory volume in one second.

Supplementary Table S4. Dietary fiber and nutrient intake according to the presence of a new airflow limitation and sex at the 5-year follow-up.

Nutrients	Total	Male, *n* = 517			Female, *n* = 922		
		Airflow Limitation (+)	Airflow Limitation (−)	*p*-Value*	Airflow Limitation (+)	Airflow Limitation (−)	*p*-Value*
	n = 1439	*n* = 41	*n* = 476		*n* = 7	*n* = 915	
Fiber, g	6.36 (0.04)	6.25 (0.26)	6.47 (0.07)	0.370	5.46 (0.11)	6.32 (0.05)	<0.001
Vitamin A, RE	806.52 (7.23)	817.68 (47.08)	815.68 (13.11)	0.966	777.70 (79.48)	801.48 (8.84)	0.815
Vitamin B1, mg	0.97 (0.01)	1.06 (0.04)	1.07 (0.01)	0.786	0.89 (0.10)	0.91 (0.01)	0.783
Vitamin B2, mg	1.01 (0.01)	1.14 (0.06)	1.08 (0.01)	0.250	1.05 (0.15)	0.97 (0.01)	0.488
Niacin, mg	13.2 (0.10)	16.17 (0.61)	15.47 (0.16)	0.227	10.89 (0.46)	11.90 (0.10)	0.070
Vitamin B6, mg	1.71 (0.01)	1.92 (0.07)	1.88 (0.02)	0.504	1.39 (0.07)	1.62 (0.01)	0.106
Folic acid, ug	249.18 (1.70)	245.93 (10.30)	251.64 (2.79)	0.568	214.53 (7.03)	248.31 (2.19)	0.002
Vitamin E, mg	13.2 (0.09)	14.52 (0.56)	13.96 (0.16)	0.325	11.49 (0.69)	12.76 (0.11)	0.331
Vitamin C, mg	113.10 (1.55)	97.49 (8.65)	110.07 (2.47)	0.153	89.49 (11.72)	115.56 (2.03)	0.262
Protein, g	62.26 (0.39)	74.41 (2.77)	70.78 (0.68)	0.139	53.24 (2.52)	57.35 (0.41)	0.383
Carbohydrate, g	228.21 (1.37)	247.61 (6.93)	249.42 (2.22)	0.818	183.25 (16.17)	216.65 (1.66)	0.080
Lipid, g	45.58 (0.36)	55.48 (3.13k)	51.40 (0.66)	0.093	40.26 (3.53)	42.14 (0.38)	0.665
Cholesterol, mg	336.11 (2.57)	367.93 (27.49)	362.97 (7.22)	0.848	365.13 (24.60)	320.49 (4.48)	0.384
Total calories	1766.56 (6.32)	2018.34 (24.64)	2042.05 (6.87)	0.334	1541.06 (25.96)	1613.68 (3.53)	0.073
**Difference between 2012 and 2017**							
Fiber, g	−2.09 (0.05)	−2.80 (0.30)	−2.10 (0.07)	0.009	−3.91 (0.74)	−2.03 (0.06)	0.004
Vitamin A, RE	−291.26 (8.76)	−384.83 (44.91)	−323.84 (14.88)	0.245	−439.99 (172.09)	−268.98 (11.08)	0.180
Vitamin B1, mg	−0.18 (0.01)	−0.30 (0.06)	−0.20 (0.01)	0.036	−0.17 (0.09)	−0.17 (0.01)	0.992
Vitamin B2, mg	−0.17 (0.01)	−0.23 (0.04)	−0.17 (0.01)	0.279	−0.07 (0.05)	−0.16 (0.01)	0.096
Niacin, mg	−2.96 (0.09)	−3.29 (0.54)	−2.88 (0.18)	0.512	−5.04 (1.14)	−2.97 (0.11)	0.105
Vitamin B6, mg	−0.45 (0.01)	−0.56 (0.07)	−0.46 (0.02)	0.165	−0.71(0.10)	−0.43 (0.01)	0.082
Folic acid, ug	−76.99 (1.93)	−102.82 (9.74)	−82.80 (3.17)	0.074	−152.15 (31.26)	−72.24 (2.47)	0.005
Vitamin E, mg	−2.50 (0.10)	−2.84 (0.55)	−2.66 (0.18)	0.777	−4.57 (0.98)	−2.38 (0.13)	0.132
Vitamin C, mg	−38.36 (1.85)	−58.48 (10.88)	−38.55 (2.86)	0.052	−87.00 (37.14)	−37.00 (2.42)	0.073
Protein, g	−11.23 (0.38)	−12.25 (2.40)	−10.79 (0.72)	0.566	−13.96 (3.35)	−11.40 (0.44)	0.615
Carbohydrate, g	−32.25 (1.42)	−31.08 (8.84)	−28.57 (2.46)	0.775	−45.36 (16.55)	−34.12 (1.79)	0.584
Lipid, g	−4.73 (0.36)	−4.76 (2.21)	−4.53 (0.71)	0.927	−5.69 (2.53)	−4.82 (0.41)	0.853
Cholesterol, mg	18.56 (3.88)	4.77 (21.93)	25.72 (7.59)	0.432	113.14 (23.41)	14.73 (4.54)	0.059
Total calories	−51.81 (1.54)	−76.53 (10.38)	−78.53 (4.05)	0.887	−41.94 (7.52)	−36.87 (0.75)	0.557

* Compared between groups with and without new airflow limitation. All values are expressed as a mean (standard error), unless otherwise stated. Abbreviation: RE, retinol equivalents.

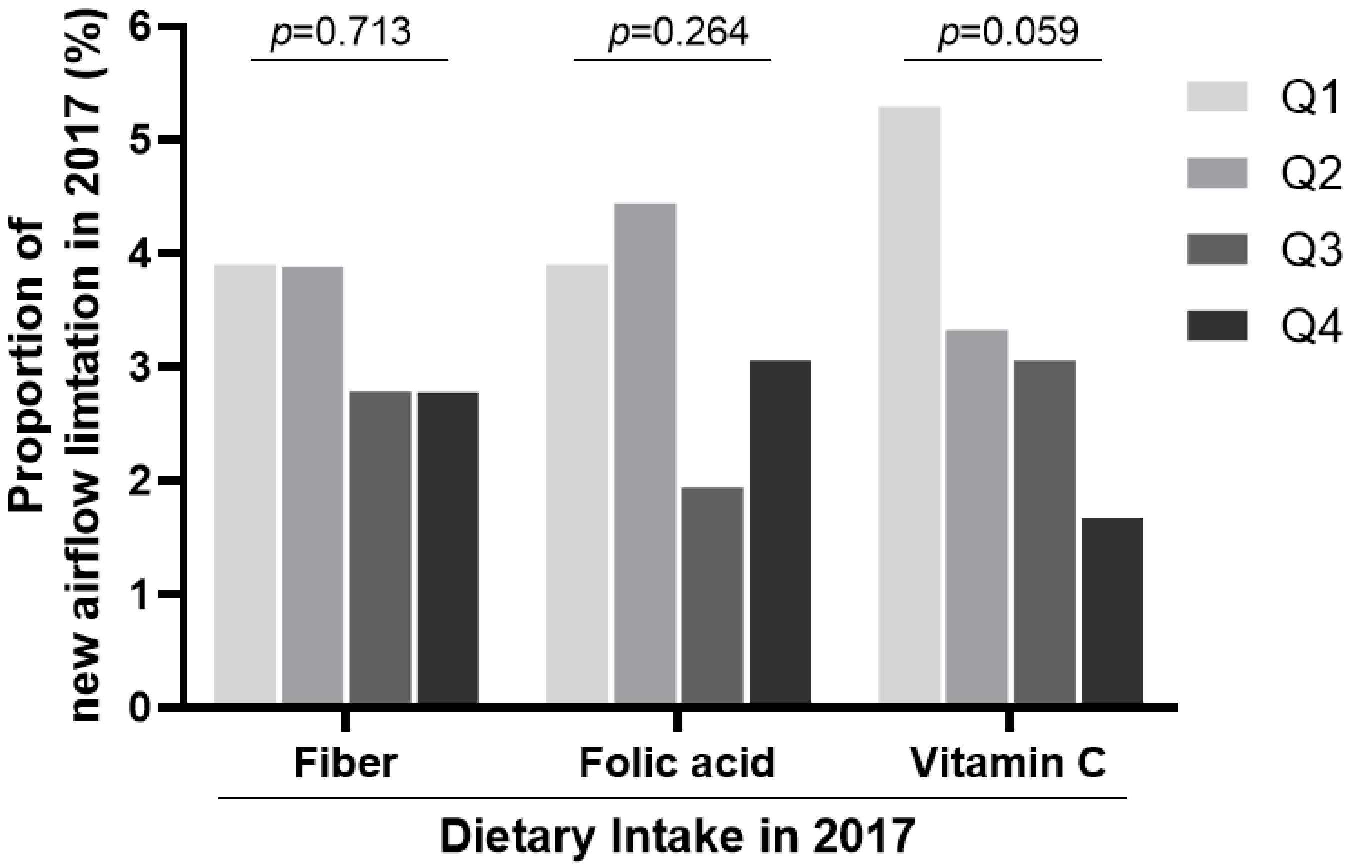

Supplementary Figure S1. Proportion of subjects with a new airflow limitation according to dietary intake quartile at the 5-year follow up (2017). The dietary intakes quartiles were as follows: fiber Q1,1.736-5.253: fiber Q2, 5.254-6.318: fiber Q3, 6.319-7.378: fiber Q4, 7.379-12.430; folic acid Q1, 44.700-202.618: folic acid Q2, 202.619-244.567: folic acid Q3, 244.567-291.011: folic acid Q4, 291.011-510.266; vitamin C Q1, 11.507-70.773: vitamin C Q2, 70.774-100.201: vitamin C Q3, 100.202-137.363: vitamin C Q4, 137.364-429.755.

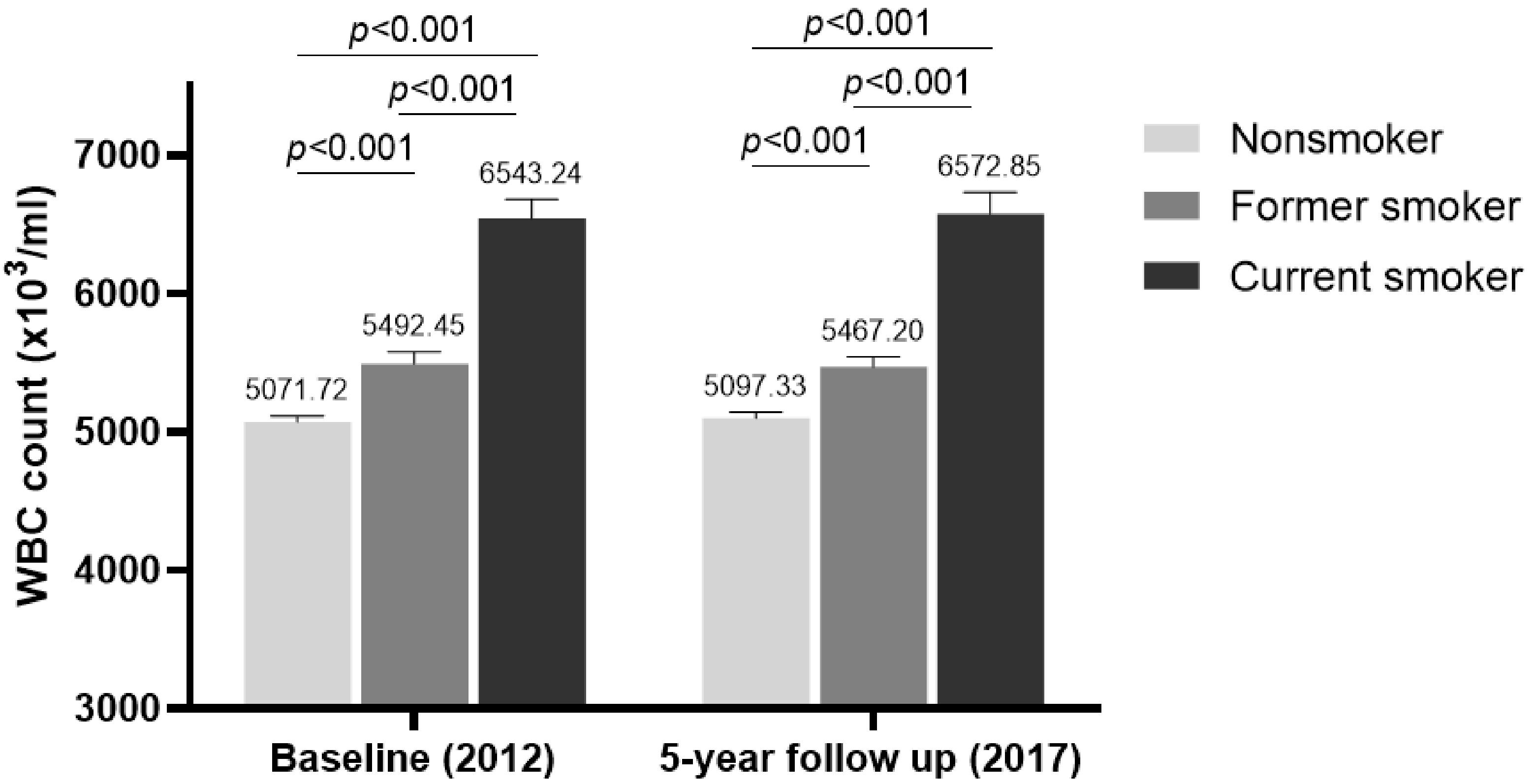

Supplementary Figure S2. WBC counts according to smoking history. WBC counts were significantly higher in current and former smokers than in nonsmokers. These results were consistent at both baseline (2012) and the 5-year follow-up (2017).

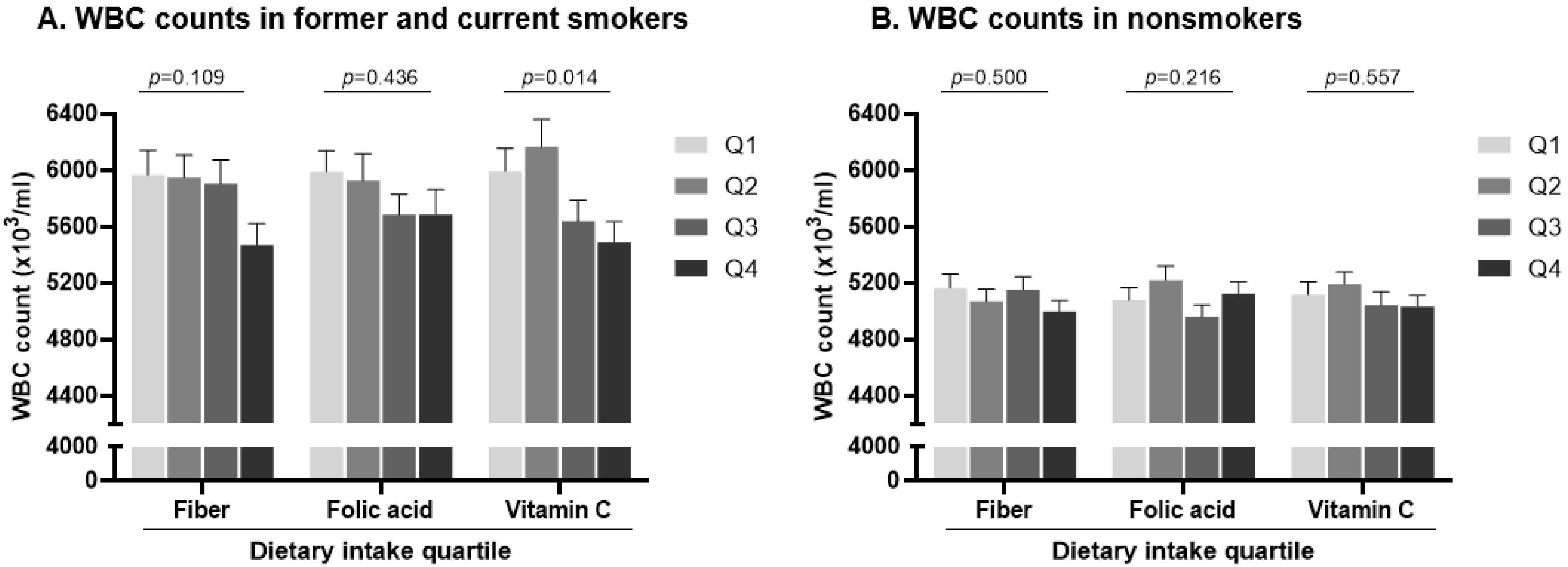

Supplementary Figure S3. WBC counts according to dietary intake quartile by subgroup analysis of smoking history. (**A**) WBC counts in former and current smokers (**B**) WBC counts in nonsmokers. As the intake of vitamin C increased, the WBC counts significantly decreased in participants with a smoking history. This trend was similar for fiber and folic acid, although it did not reach statistical significance. Abbreviation: WBC, white blood cell. The dietary intakes quartiles in former and current smokers were as follows: fiber Q1, 2.686-5.272: fiber Q2, 5.273-6.349: fiber Q3, 6.350-7.431: fiber Q4, 7.432-12.430; folic acid Q1, 98.876-203.263: folic acid Q2, 202.264-246.628: folic acid Q3, 246.629-292.763: folic acid Q4, 292.764-510.266; vitamin C Q1, 21.732-72.782: vitamin C Q2, 72.783-103.658: vitamin C Q3, 103.659-142.299: vitamin C Q4, 142.300-429.755. The dietary intakes quartiles in non-smokers were as follows: fiber Q1, 2.686-5.272: fiber Q2, 5.273-6.349: fiber Q3, 6.350-7.431: fiber Q4, 7.432-12.430; folic acid Q1, 98.876-203.263: folic acid Q2, 202.264-246.628: folic acid Q3, 246.629-292.763: folic acid Q4, 292.764-510.266; vitamin C Q1, 21.732-72.782: vitamin C Q2, 72.783-103.658: vitamin C Q3, 103.659-142.299: vitamin C Q4, 142.300-429.755.

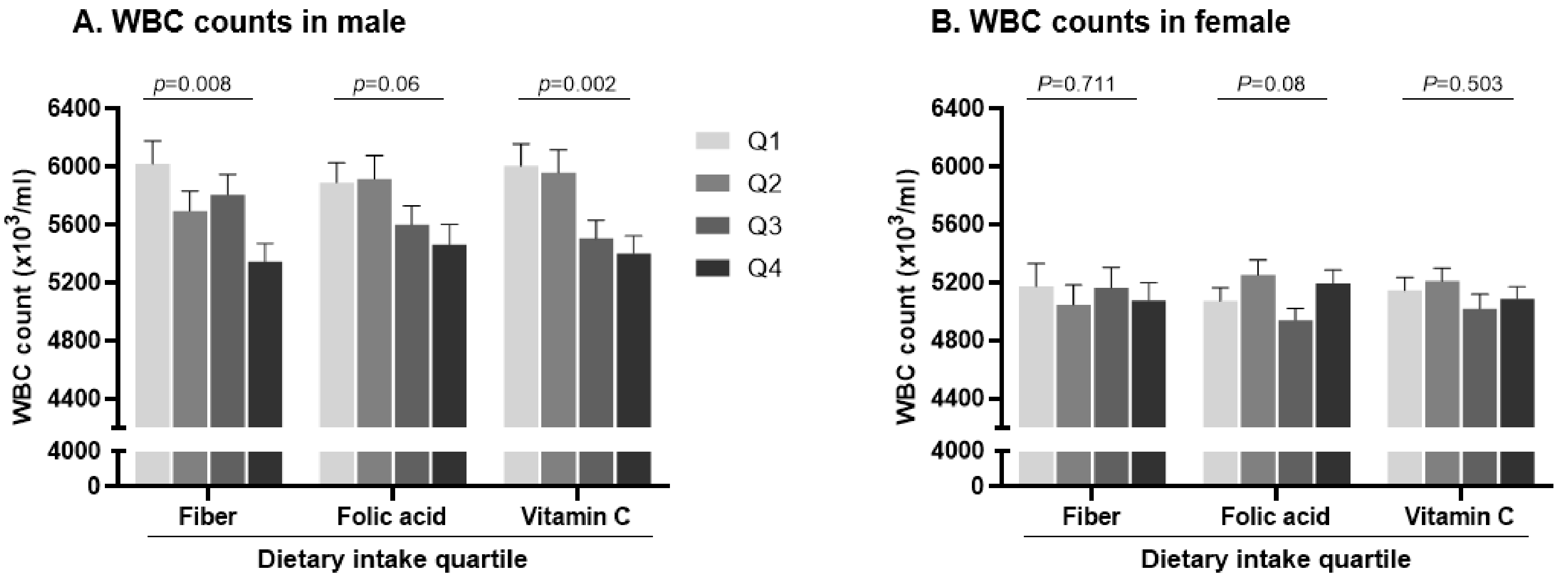

Supplementary Figure S4. WBC counts according to dietary intake quartile by subgroup analysis of sex. (**A**) WBC counts in male (**B**) WBC counts in female. As the intake of fiber and vitamin C increased, the WBC counts significantly decreased in male subjects. This trend was not noted in female subjects in which the WBC counts were similar in all quartile**.** Abbreviation: WBC, white blood cell. The dietary intakes quartiles in male were as follows: fiber Q1, 1.736-5.391: fiber Q2, 5.392-6.427: fiber Q3, 6.428-7.445: fiber Q4, 7.446-11.348; folic acid Q1, 44.700-208.214: folic acid Q2, 208.215-246.872: folic acid Q3, 246.873-288.408: folic acid Q4, 288.409-456.584; vitamin C Q1, 11.507-70.417: vitamin C Q2, 70.418-99.497: vitamin C Q3, 99.498-130.955: vitamin C Q4, 130.956-405.787. The dietary intakes quartiles in female were as follows: fiber Q1, 2.647-5.121: fiber Q2, 5.122-6.230: fiber Q3, 6.321-7.338: fiber Q4, 7.339-12.430; folic acid Q1, 98.877-199.072: folic acid Q2, 199.073-243.236: folic acid Q3, 243.237-292.021: folic acid Q4, 292.021-510.266; vitamin C Q1, 24.813-70.960: vitamin C Q2, 70.961-100.668: vitamin C Q3, 100.669-141.466: vitamin C Q4, 141.467-429.755.
